# Liquorice ingestion attenuates vasodilatation via exogenous nitric oxide donor but not via β_2_-adrenoceptor stimulation

**DOI:** 10.1371/journal.pone.0223654

**Published:** 2019-10-18

**Authors:** Elina J. Hautaniemi, Antti J. Tikkakoski, Arttu Eräranta, Mika Kähönen, Esa Hämäläinen, Ursula Turpeinen, Heini Huhtala, Jukka Mustonen, Ilkka H. Pörsti

**Affiliations:** 1 Faculty of Medicine and Health Technology, Tampere University, Tampere, Finland; 2 Department of Clinical Physiology, Tampere University Hospital, Tampere, Finland; 3 HUSLAB, Helsinki University Hospital, Helsinki, Finland; 4 Department of Clinical Chemistry, Biomedicum, Helsinki University, Helsinki, Finland; 5 Faculty of Social Sciences, Tampere University, Tampere, Finland; 6 Department of Internal Medicine, Tampere University Hospital, Tampere, Finland; Kurume University School of Medicine, JAPAN

## Abstract

We examined the effect of liquorice ingestion on haemodynamic responses to exogenous nitric oxide donor (nitroglycerin) and β_2_-adrenoceptor agonist (salbutamol), and 11β-hydroxysteroid dehydrogenase activity, in 21 volunteers and 21 reference subjects. Haemodynamic data was captured before and after sublingual nitroglycerin (0.25 mg) and inhaled salbutamol (400 μg) during orthostatic challenge utilising radial pulse wave analysis and whole-body impedance cardiography. The recordings were performed at baseline and following two weeks of liquorice intake (290–370 mg/d glycyrrhizin). Urinary cortisone and cortisol metabolites were examined. Liquorice intake elevated aortic systolic and diastolic blood pressure and systemic vascular resistance when compared with the reference group. Following research drug administration the liquorice-induced increase in systemic vascular resistance was observed in the presence of nitroglycerin (p<0.05) but no longer in the presence of salbutamol. Liquorice ingestion decreased cardiac chronotropic response to upright posture (p = 0.032) in unadjusted analysis, but when adjusted for age and sex the difference in the upright change in heart rate was no longer significant. The urinary cortisone to cortisol metabolite ratio decreased from 0.70 to 0.31 (p<0.001) after liquorice intake indicating significant inhibition of the 11β-hydroxysteroid dehydrogenase type 2. In the reference group the haemodynamic variables remained virtually unchanged. These results suggest that liquorice exposure impaired vasodilatation *in vivo* that was induced by exogenous nitric oxide donor but not that induced by β_2_-adrenoceptor stimulation.

**Trial registration:** EU Clinical Trials Register 2006-002065-39

ClinicalTrials.gov NCT01742702.

## Introduction

Hypertension and hypokalemia-induced secondary disorders are the main complications of liquorice ingestion reported in the literature [1]. Commercial liquorice extract is mainly obtained from the root of Glycyrrhiza glabra that is cultivated in temperate and semi-tropical parts of Europe and Asia [1,2]. The clinical effects of liquorice are mediated via inhibition of the enzyme 11β-hydroxysteroid dehydrogenase type 2 (11β-HSD2) by the active metabolite glycyrrhetinic acid (GA), which leads to impaired conversion of active cortisol to inactive cortisone [1,3]. Increased cellular levels of cortisol bind both to the mineralocorticoid receptor (MR) and the glucocorticoid receptor (GR) [1,3], while increased activation of both of these receptors can cause hypertension [4,5]. Normally aldosterone is the physiological agonist of the MR due to the protective effect of enzyme 11β-HSD2 [6,7], but following liquorice intake the action of cortisol is enhanced [1,3].

In the distal nephron, excessive MR activation by cortisol results in sodium and water retention, elevated blood pressure (BP), decreased plasma potassium concentration and suppression of the renin-angiotensin-aldosterone system [8]. The MR, GR and 11β-HSD2 are also expressed in the vascular wall, in both endothelial cells (EC) and the vascular smooth muscle cells (VSMC) [9–11]. Glucocorticoids may increase vascular tone via alterations within the endothelium including increased release of vasoconstrictors like angiotensin II and endothelin (ET)-1, and impaired endothelium-derived relaxation via nitric oxide (NO), or by direct actions within the VSMC including up-regulation of angiotensin II receptors [9]. MR antagonism has been found to reduce pulse wave velocity, a marker of arterial stiffness, in hypertensive subjects [12]. Both the MR and GR are found in cardiomyocytes, while the expression of 11β-HSD2 in the heart may be restricted to the coronary vasculature [13]. However, one report proposed the presence of 11β-HSD2 in human cardiac tissue [14]. Results from transgenic mouse models suggest that MR activation in cardiomyocytes contributes to progression of cardiac disease, while GR signalling is crucial for the maintenance of normal cardiac function [15,16].

Previously, we found that liquorice intake for two weeks increased BP via volume expansion, elevated peripheral arterial resistance and increased large arterial stiffness [17]. We also observed reduced chronotropic response and enhanced central wave reflection in the upright posture after liquorice exposure [17]. To examine the mechanisms underlying the elevation of systemic arterial resistance, here we investigated possible alterations in vasodilatory responses induced by exogenous NO donor and β_2_-adrenoceptor agonist after liquorice exposure in supine and upright positions. To evaluate the suppression of enzyme 11β-HSD2 activity, the urinary ratio of cortisone to cortisol metabolites was examined [8].

## Methods

### Ethical statement

All participants gave written informed consent, and the investigation conformed to the Declaration of Helsinki. The study was approved by the Ethics Committee of Tampere University Hospital on the 3^rd^ of April 2007 (study code R07053M). The study is registered in the database of clinical trials (ClinicalTrials.gov, ID: NCT01742702), and is a part of an on-going investigation on haemodynamics (DYNAMIC-study code R06086M, EudraCT-number 2006-002065-39).

### Study subjects and design

The study protocol (in Finnish) with an English summary have been published [18], and the CONSORT checklist is presented as supporting information (S1 CONSORT checklist). The recruitment and characteristics of the subjects in the liquorice group and the study design were reported elsewhere [17,18]. The present results originate from the same liquorice intervention from which we published results concerning liquorice-induced changes in haemodynamics in the supine position [18] and during orthostatic challenge [17].

In the present study, the focus was on the mechanisms of vasodilatation induced by exogenous NO (nitroglycerin) and β_2_-adrenoceptor stimulation (salbutamol) after 2 weeks of liquorice intake. The intervention group was the same as in earlier reports [17,18], while all findings concerning NO and β_2_-adrenoceptor-induced vasodilatation are novel, and the reference group is new. The participants were recruited via announcements distributed at the Tampere University, Tampere University Hospital, and occupational health care units, and through notices in a local newspaper, and Varala Sports Institute. The study data were collected from May 2007 to June 2012 at the Departments of Internal Medicine and Clinical Physiology, Tampere University Hospital, Tampere, Finland. Medical examination was performed by a physician and the participants were interviewed for lifestyle habits and medical history [17,18]. Blood and urine samples were collected after ~12 hours of fasting and standard 12-lead electrocardiograms were recorded.

**Fig 1:**
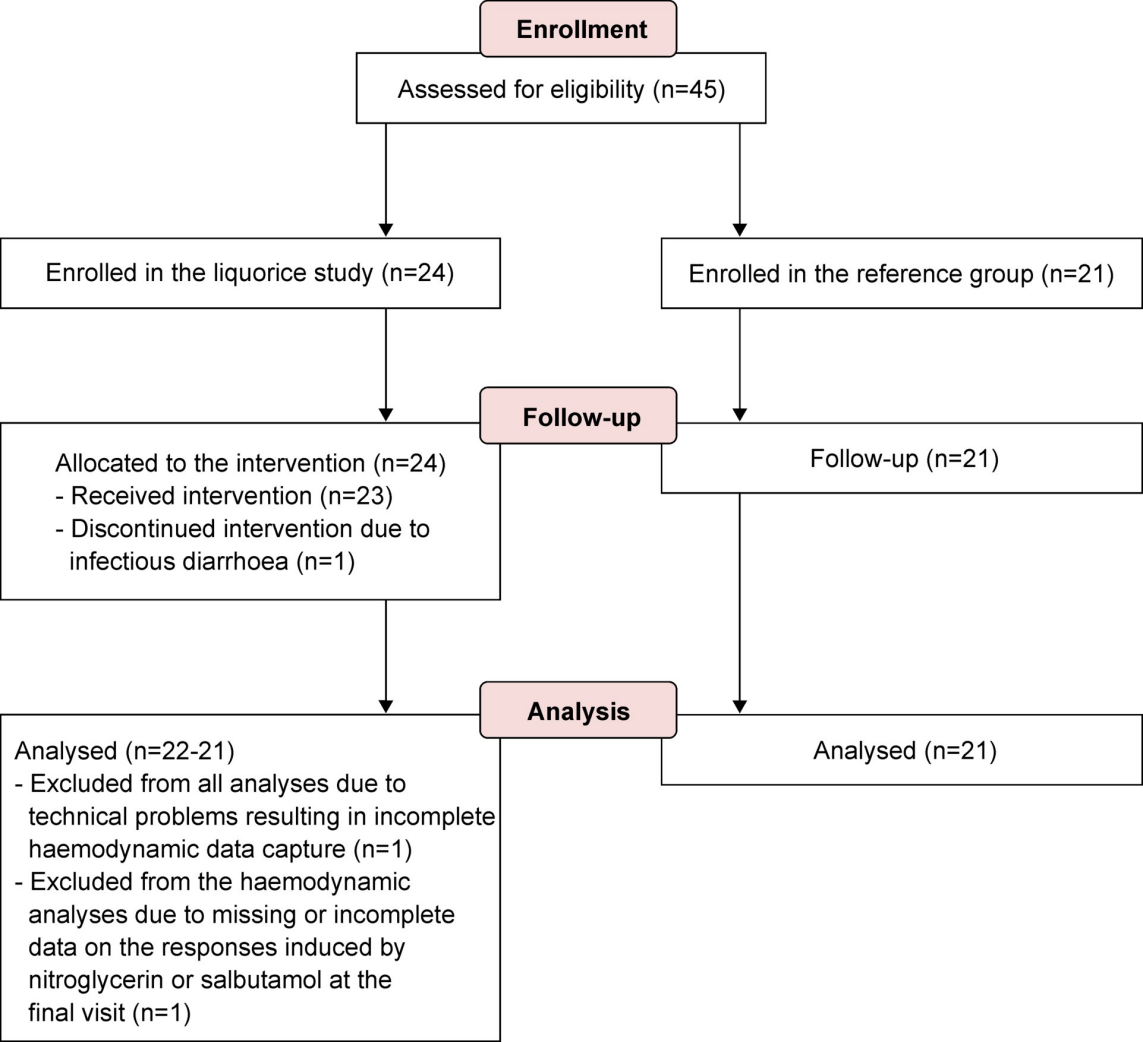
The CONSORT flow diagram of study participants.

The final liquorice group included 22 healthy volunteers (14 women and 8 men) who consumed liquorice for two weeks [17]. Participant age ranged 23–58 years and the supine mean (standard deviation) (SD) brachial office BP was 119/70 (8/7) mmHg. Subjects with office BP > 140/90 mmHg or cardiovascular disease with drug therapy were excluded [17,18]. In addition, pregnancy or consumption of liquorice >300 grams per week excluded participation [17,18]. One male subject in the liquorice group did not receive sublingual nitroglycerin at the final visit due to nitroglycerin-induced headache after baseline measurements. Haemodynamic recordings were incomplete in one female subject after salbutamol inhalation at the final visit. Therefore, the statistical analyses of the head-up tilt table test included 22 subjects from visit 1 and 21 subjects from visit 2, while the analyses of laboratory values included all of the 22 subjects (Fig 1). The medications of the subjects in the liquorice group were reported elsewhere [17].

In order to distinguish haemodynamic changes induced by repeated measurements from the liquorice-induced changes, we investigated 21 volunteers maintaining their habitual diet. This group consisted of 3 women and 18 men, age 36–56 years with baseline supine mean (SD) brachial office BP 134/85 (9/7) mmHg. The reference subjects participated in our study on haemodynamics (DYNAMIC study, R06086M), and they were chosen so that all were without BP lowering medication, heart disease, diabetes, and cerebrovascular or peripheral arterial disease. The following clinical conditions with stable medications were present in the reference group: statin treatment for hypercholesterolemia (n = 3); proton pump inhibitor for gastro-oesophageal reflux (n = 2); selective serotonin reuptake inhibitor for depression (n = 2); thyroxine substitution (n = 1); intramuscular vitamin B_12_ replacement (n = 1); oral progestin contraceptive (n = 1); activated protein-C resistance without medication (n = 1); and cholelithiasis (n = 1). The liquorice group contained 4 present smokers and one previous smoker, while the reference group contained no present smokers and 9 previous smokers.

The study design was open-label [17,18]. During the 2-week liquorice intervention, the glycyrrhizin dose was 290–370 mg/d from commercial liquorice products (Halva liquorice^TM^ or Kouvola liquorice^TM^), corresponding to liquorice consumption from 120 to 300 g/d, respectively [17,18]. The use of two liquorice products was based on the recommendation of two research group members who piloted the intervention, as this protocol prevented aversion development towards liquorice [18]. During the exposure, the average intake of carbohydrates from the two liquorice products was ~150 g/d [18]. Before baseline measurements, all liquorice products were to be avoided for 3 weeks. In the reference group, the subjects were instructed to continue their conventional eating habits, and they were not aware of acting as a reference for the liquorice group [17,18]. The reported liquorice consumption frequency among the reference group was as follows: monthly or lower (n = 17), weekly (n = 4).

Haemodynamics were recorded before and after two weeks of liquorice exposure, and before and after 3 weeks (n = 16) or 10 months of follow-up (n = 5) in the reference group. For cortisone and cortisol metabolite analyses, 24-hour urine was collected at weeks 0 and 2 in the liquorice group.

### Laboratory analyses

Blood count was determined using ADVIA 120 or 2120 (Bayer Health Care, Tarrytown, NY, USA), and plasma sodium, potassium, creatinine, glucose, triglyceride, and total, high-density, and low-density lipoprotein cholesterol concentrations were determined using Cobas Integra 700/800 (F. Hoffmann-LaRoche Ltd, Basel, Switzerland). Since plasma creatinine was within the normal range, estimated glomerular filtration rate (GFR) was calculated with the RULE formula [19]. Plasma aldosterone was measured by radioimmunoassay (Aldosterone RIA Test DSL-8600, Diagnostics Systems Laboratories Inc, Webster, TX, USA) and renin activity utilising GammaCoat Plasma Renin Activity assay (Diasorin). Urinary tetrahydrocortisone (THE) and allo-tetrahydrocortisol plus tetrahydrocortisol (allo-THF+THF) concentrations were analysed using liquid chromatography-tandem mass spectrometry as described previously [20].

### Pulse wave analysis

Radial BP and pulse wave form were continuously recorded by a tonometric sensor from the left radial pulsation (Colin BP-508T, Colin Medical Instruments Corp., San Antonio, Texas, USA). Contralateral brachial BP measurements were used to calibrate the radial BP recordings approximately every 2.5 min [21,22]. Continuous aortic BP was derived from the radial signal with the SphygmoCor PWMx pulse wave monitoring system (Atcor Medical, Australia) [23]. Augmentation index (AIx) (augmentation pressure/pulse pressure*100) was determined from the aortic pulse wave form.

### Whole-body impedance cardiography

Beat-to-beat heart rate (HR), stroke volume, cardiac index (cardiac output/body surface area), pulse wave velocity (PWV) and extracellular water volume were measured with a whole-body impedance cardiography device (CircMon^R^, JR Medical Ltd., Tallinn, Estonia) as previously described [24–26]. Systemic vascular resistance index (systemic vascular resistance/body surface area) (SVRI) was calculated from the tonometric BP and cardiac index recorded by CircMon^R^. The stroke volume and cardiac output values determined with the CircMon^R^ show good correlation with values obtained using 3-dimensional echocardiography [27] and the thermodilution method [24,25]. In addition, the reproducibility and repeatability of the measurements are good [21,22].

Aortic-to-popliteal PWV was calculated by measuring the time difference between the onset of the decrease in impedance in the whole-body impedance signal and the popliteal artery signal with the CircMon^R^ software [26]. The recorded PWV values show excellent correlation with values measured using either ultrasound or the tonometric SphygmoCor method [26,28].

### Haemodynamic measurement protocol

The recordings were performed in a quiet, temperature-controlled laboratory by research nurses [21,22]. Prior to the measurements, caffeine products, smoking and heavy meal for ≥4 hours and alcohol for ≥24 hours were precluded. The electrodes for impedance cardiography were placed on the body surface, the tonometric sensor on the left radial artery pulsation, and a BP cuff to the right upper arm [21,22]. The left arm was abducted to 90 degrees and in an arm support at the level of the heart.

The beat-to-beat haemodynamic data was captured during six consecutive 5-min periods [22]. The 0.25 mg sublingual nitroglycerin resoriblet (Nitro resoriblet; Orion Pharma, Espoo, Finland) and 400 μg salbutamol inhalation (Ventoline; GlaxoSmithKline, Uxbridge, Middlesex, UK) were administered on the same day of measurements with at least one hour intermission between the drugs. Salbutamol was delivered by the use of a spacer device (Volumatic; Allen & Hanbury’s, Uxbridge, Middlesex, UK) after guidance and supervision by the research nurse. The total recording time was 2 x 30 min: the subjects were first resting supine on the tilt table for 5 min, followed by 5 min of head-up tilt to >60 degrees, and then the tilt table was restored to supine position. Then the research drug (nitroglycerin or salbutamol) was administered and the protocol (5 min supine—5 min upright—5 min supine) was repeated [22].

As the action of inhaled salbutamol begins from the first minute with stable plasma concentrations 5–20 minutes after administration [29], the measurement period of 15 min is sufficient to detect salbutamol-induced changes in haemodynamics. If the subject reported presyncopal symptoms and BP fell progressively during nitroglycerin-stimulated orthostatic challenge, the tilt table was restored to supine position before the 5 min was completed [30].

### Statistical analyses

Means of the beat-to-beat values of each minute of the 30-min recordings were calculated and used in the statistics. If the head-up tilt was aborted after sublingual nitroglycerin before 5 min was completed, the missing upright values were replaced by the preceding values, providing that at least 2 min of upright recordings were available. Statistical analyses were performed using IBM SPSS Statistics Version 24 (IBM Corporation, Armonk, NY, USA). Variable values are reported as means with SDs, standard errors of the mean or 95% confidence intervals. The Shapiro-Wilk test and histograms were used to check the normal distributions of the variables.

To detect a 8 mmHg difference in the change in systolic BP from baseline (two-tailed alpha level 0.05, 80% power, SD of 9, power analysis method for two-sample t-test), the required minimum sample size was 20 controls and 20 experimental subjects. The area under the curve (AUC) was calculated utilising the IBM SPSS Statistics Version 24 [31] for every 5-min period in the supine and upright position during the recording (for aortic systolic and diastolic BP, HR, AIx, cardiac index, and SVRI), in both the absence and presence of the research drugs. Since there were significant differences in several variables between the liquorice and reference group at baseline (see below), the differences in the AUCs between visit 1 and visit 2 in the liquorice versus reference group were compared by the use of analysis of covariance. In order to avoid excessive loss of statistical power resulting from adjustment for multiple covariates [32,33], the outcomes were controlled for age [34] and sex [35], as these were considered the most important confounding factors. Wilcoxon signed-rank test was utilised to examine the within-group changes in urinary cortisone and cortisol metabolites, plasma aldosterone, potassium, and renin activity, and PWV. The mean supine values of PWV were calculated from the minutes 3–5 of the recordings when the signal was most stable. Non-continuous variables were analysed using Chi-square test. *P*<0.05 was considered statistically significant.

## Results

### Characteristics of the study subjects at visit 1

The demographic and laboratory characteristics at baseline are shown in Table 1. The study groups differed in sex distribution and age: the majority of the participants in the reference group were men (18/3) and mean age was 12 years higher compared to the liquorice group. In addition, BMI, plasma fasting triglycerides, low density lipoprotein, glucose, haemoglobin and sodium were higher, and high density lipoprotein lower in the reference group than in the liquorice group.

**Table 1:** Baseline characteristics of the study population.

	Reference (n = 20–21^a^)		Liquorice (n = 22)		*P* value^b^
	Mean	SD	Mean	SD	
Number (percentage) of males	18 (86%)		8 (36%)		0.002^c^
Age (years)	47.3	6.2	34.9	9.2	<0.001
Body mass index (kg/m^2^)	28.2	3.3	23.3	1.9	<0.001
Haemoglobin (g/l)	151	11	139	9	<0.001
Fasting plasma					
Cholesterol (mmol/l)	5.0	0.8	4.6	0.8	0.069
Triglycerides (mmol/l)	1.38	0.52	0.83	0.32	<0.001
High density lipoprotein (mmol/l)	1.31	0.28	1.83	0.36	<0.001
Low density lipoprotein (mmol/l)	3.2	0.7	2.4	0.6	0.001
Glucose (mmol/l)	5.5	0.5	5.2	0.4	0.008
Sodium (mmol/l)	141	1.4	140	1.3	0.001
Potassium (mmol/l)	3.8	0.3	3.9	0.3	0.264
eGFR^d^ (ml/min per 1.73 m^2^)	115	12	114	16	0.413

^a^Blood samples for fasting plasma values were not obtained from one subject.

^b^Mann-Whitney U-test

^c^Chi-square test

^d^eGFR, estimated glomerular filtration rate using the RULE formula [19].

**Fig 2:**
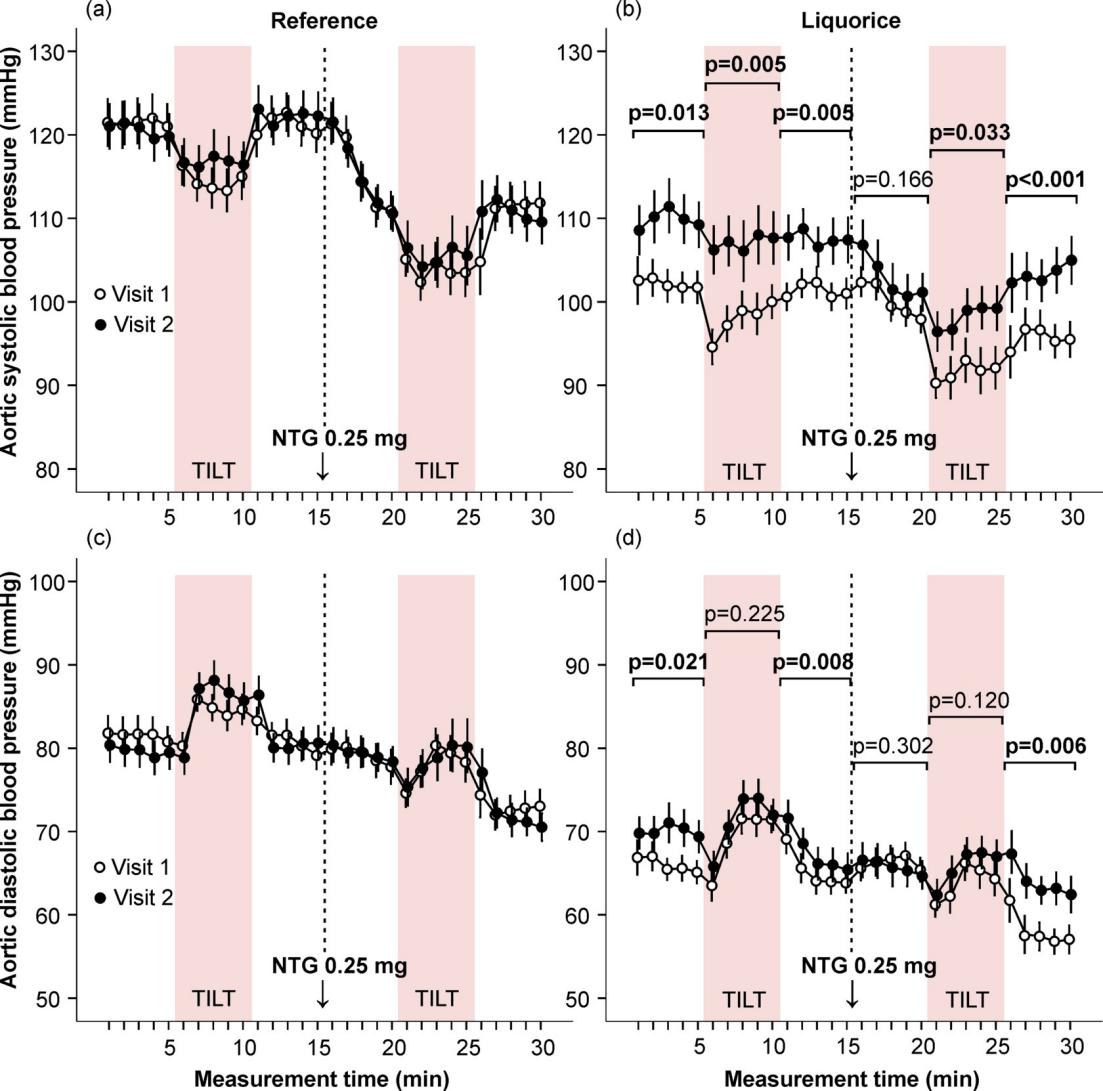
Aortic blood pressure at baseline and after the follow-up (reference group) or intervention (liquorice ingestion). Systolic (a, b) and diastolic (c, d) blood pressure were measured in the absence (0–15 min) and presence (16–30 min) of sublingual nitroglycerin during supine position and orthostatic challenge. The passive head-up tilt was carried out from 5 to 10 min, and 20 to 25 min. Mean and standard error of the mean, statistical analyses compared the difference of the area under the curve between visit 1 and visit 2 in the liquorice versus the reference group, adjusted for age and sex.

**Fig 3:**
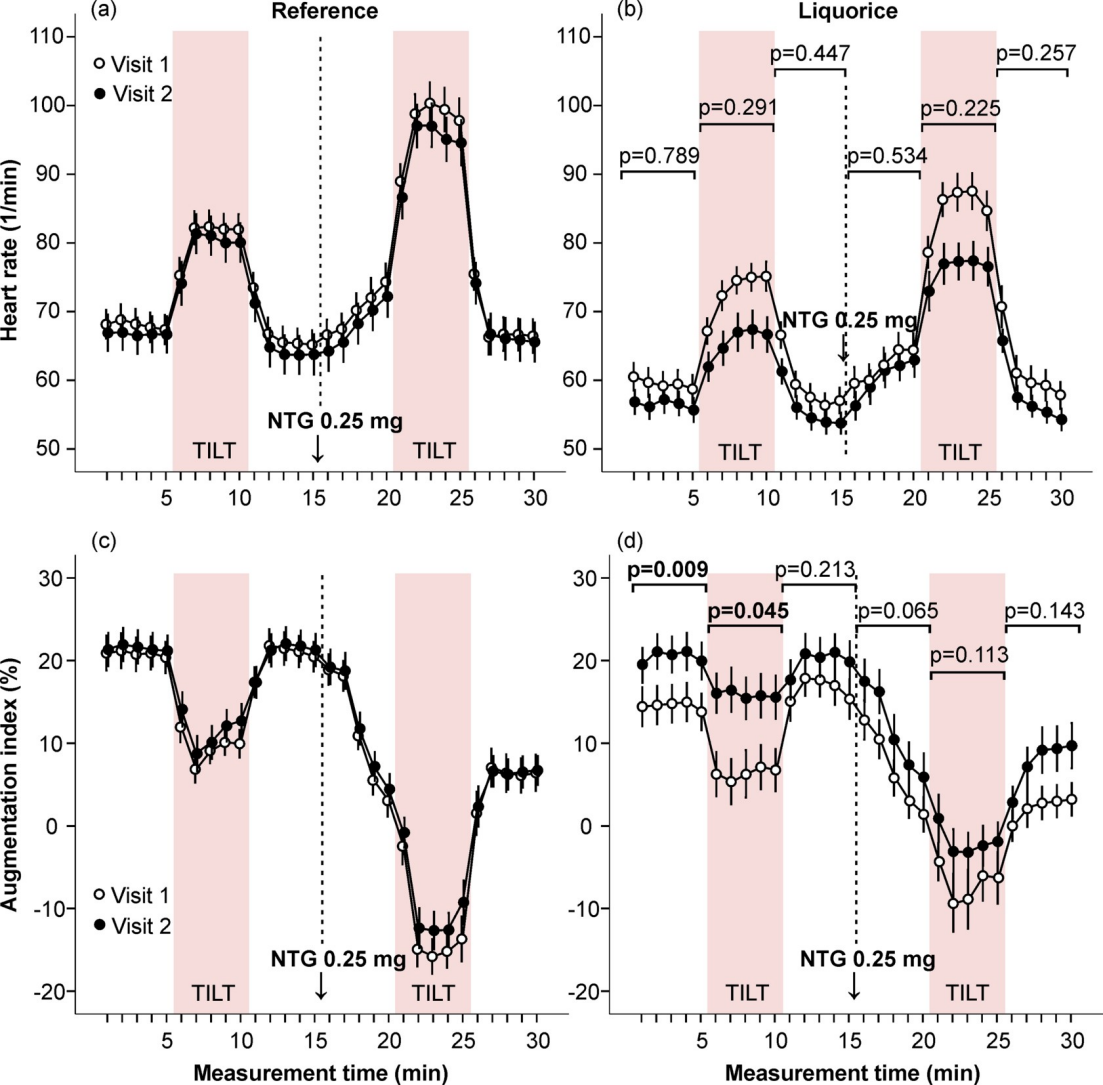
Heart rate and augmentation index in the absence and presence of sublingual nitroglycerin. Heart rate (a, b) and augmentation index (c, d) were measured at baseline and after the follow-up. Mean and standard error of the mean, the difference of the area under the curve between visit 1 and visit 2 was compared in the liquorice versus the reference group, adjusted for age and sex.

At baseline, during the initial 15-min of the recording (in the absence of research drugs) aortic systolic (p<0.05) and diastolic (p<0.01) BP (Fig 2), and HR (p<0.05, Fig 3) were higher in the reference group than in the liquorice group (univariate analysis of variance, adjusted for age and sex).

### Influences of liquorice intake at visit 2

The purpose of the reference group was only to test the hypothesis whether the recorded haemodynamic values change during repeated measurements. The reproducibility of the haemodynamic responses to upright posture during 12 weeks of follow-up has been previously shown in three parallel groups of subjects [36].

#### Renin and aldosterone levels and urinary glucocorticoid excretion

**Fig 4:**
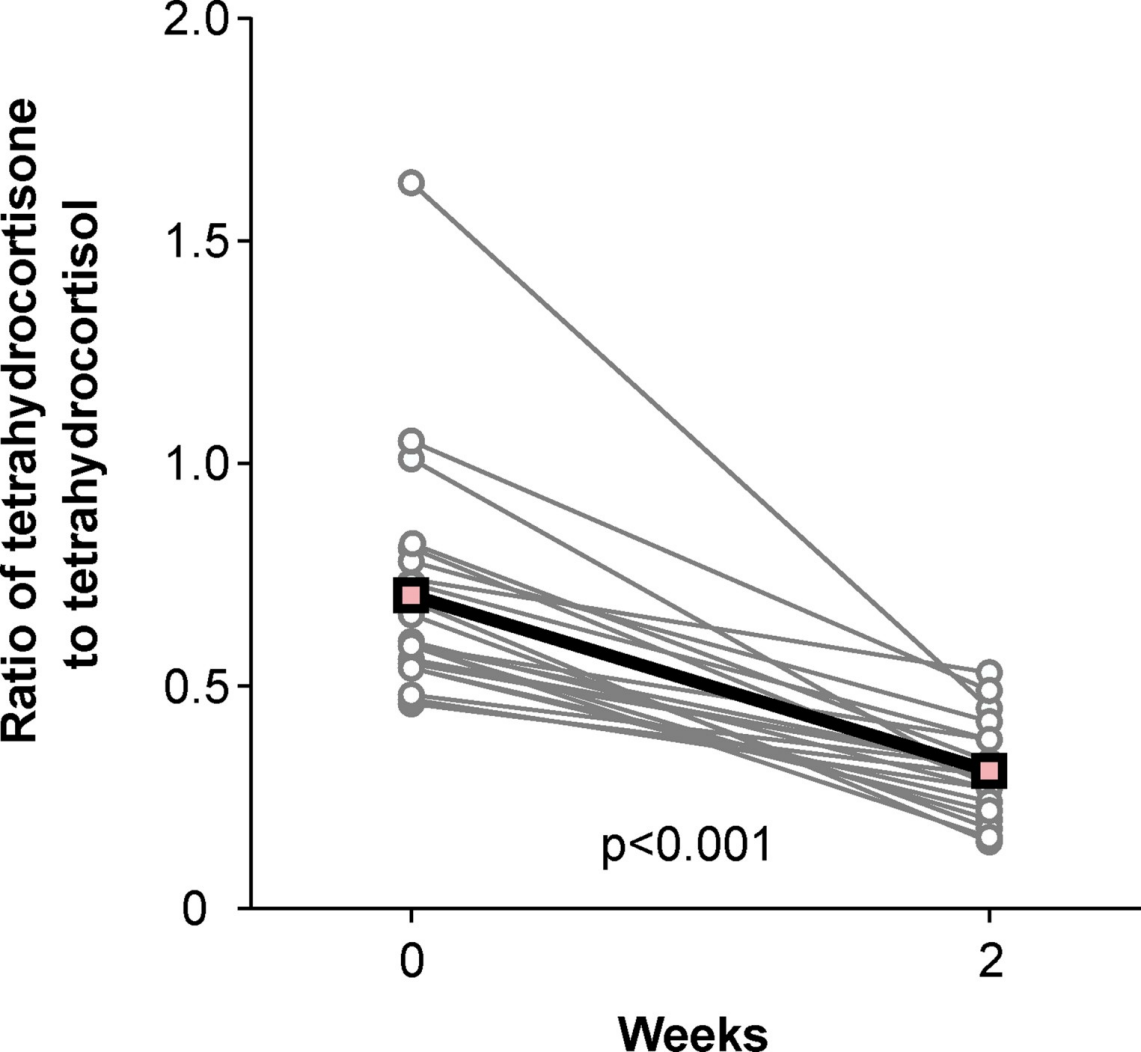
Liquorice intake and urinary glucocorticoid excretion. The urinary ratio of tetrahydrocortisone to tetrahydrocortisol at baseline and after two weeks of liquorice ingestion, grey lines depict each individual, thick black line depicts mean values, n = 22.

Two weeks of liquorice ingestion decreased plasma aldosterone concentration by -304 рmol/l (95% CI -434 to -174, p<0.001), renin activity by -1.2 μg/l/h (-2.1 to -0.3, p<0.001), and potassium concentration by -0.2 mmol/l (-0.4 to -0.1, p = 0.012). At baseline, mean (SD) urinary THE excretion was 28.3 (18.9) nmol/l and allo-THF+THF 40.0 (24.1) nmol/l, and the ratio of THE to allo-THF+THF was 0.70 (0.26). After the liquorice intervention the mean change (95% CI) in THE was -16.9 nmol/l (-25.9 to -7.9, p = 0.001) and in allo-THF+THF -4.0 nmol/l (-14.1 to 6.2, p = 0.205). The ratio of THE to allo-THF+THF was decreased by -0.39 (from 0.70 to 0.31, p<0.001) following liquorice indicating significant inhibition of 11β-HSD2 (Fig 4).

#### Pulse wave velocity

Mean values of PWV during the study are presented in Table 2. Two weeks of liquorice ingestion increased PWV (p = 0.046), while in the reference group PWV did not change (p = 0.689).

**Table 2:** Pulse wave velocity during the study. Mean values and standard deviations.

	Reference (n = 21)		Liquorice (n = 22)		*P* (ANOVA)^a^
	Mean	SD	Mean	SD	
**PWV (m/s)**					
Visit 1	8.7	1.1	7.1	0.7	0.066
Visit 2	8.7	1.2	7.4	0.8	0.625
Wilcoxon *P* value^b^	0.689		0.046		

^a^Adjusted for age and sex

^b^Comparison between visit 1 and visit 2.

#### Liquorice and haemodynamics in the absence and presence of sublingual nitroglycerin

**Fig 5:**
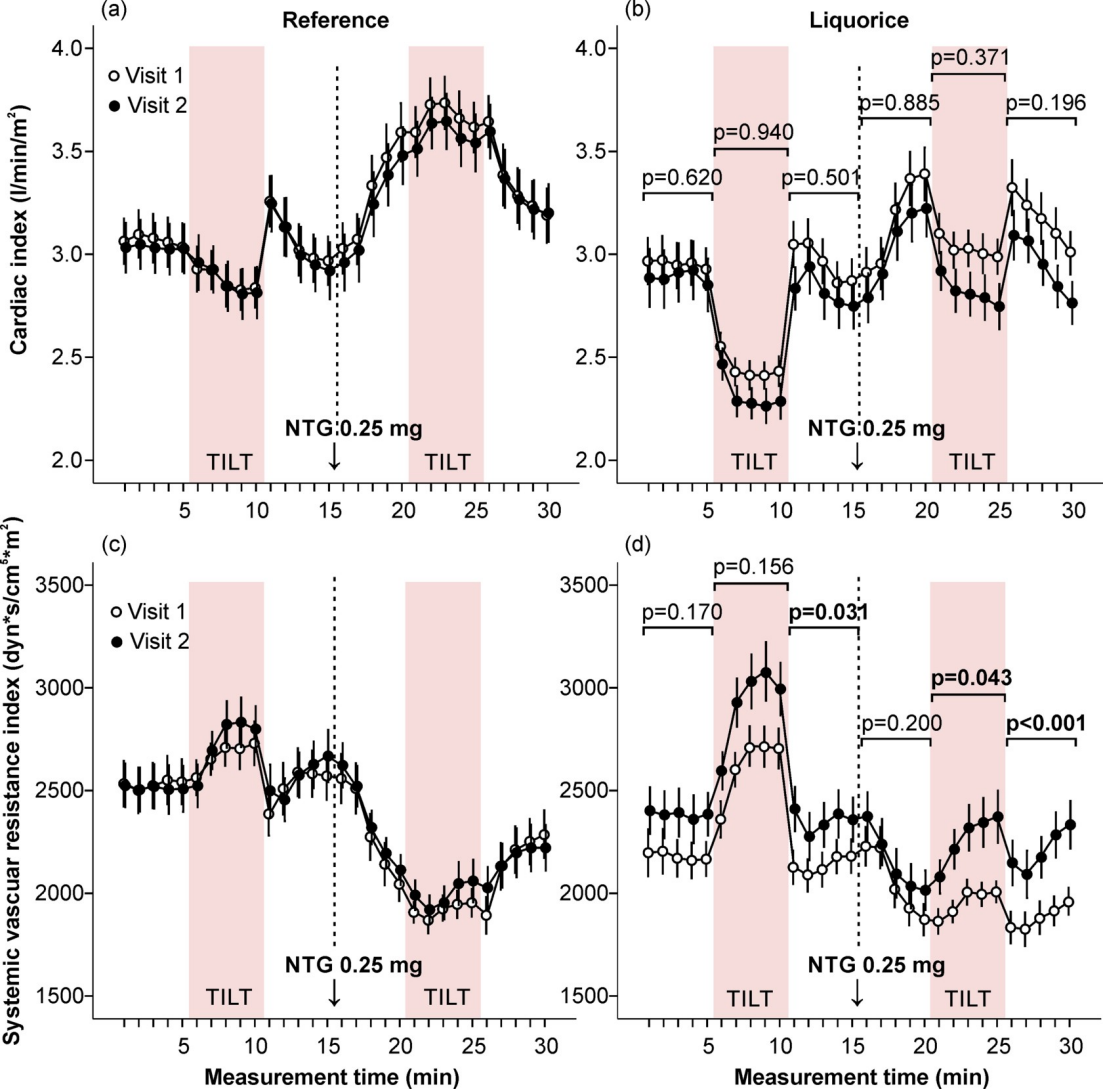
Cardiac index and systemic vascular resistance index in the absence and presence of sublingual nitroglycerin. Cardiac index (a, b) and systemic vascular resistance index (c, d) were measured at baseline and after the follow-up. Mean and standard error of the mean, the difference of the area under the curve between visit 1 and visit 2 was compared in the liquorice versus the reference group, adjusted for age and sex.

**Fig 6:**
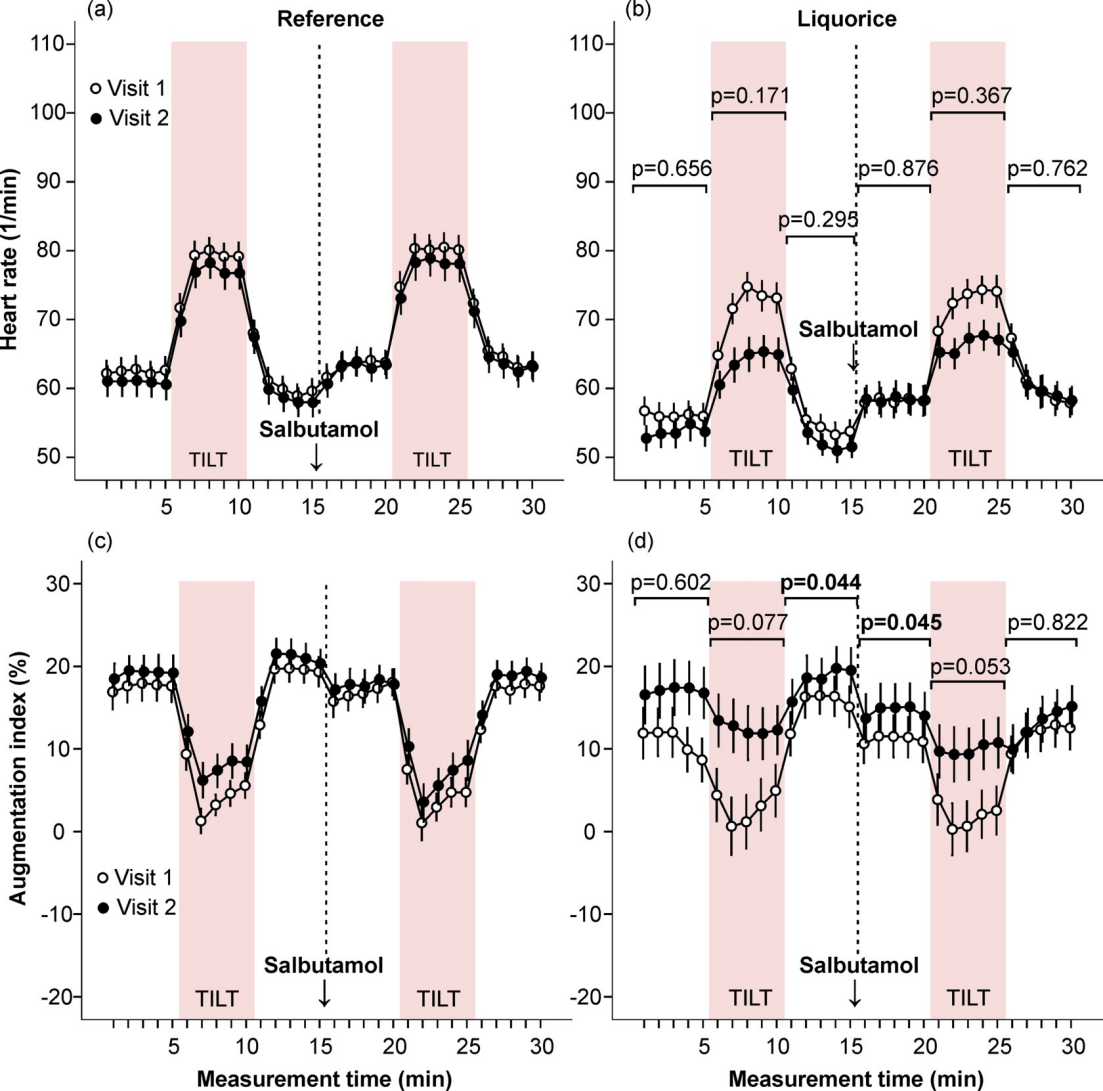
Heart rate and augmentation index in the absence and presence of salbutamol inhalation. Heart rate (a, b) and augmentation index (c, d) were measured at baseline and after the follow-up. Mean and standard error of the mean, the difference of the area under the curve between visit 1 and visit 2 was compared in the liquorice versus the reference group, adjusted for age and sex.

**Fig 7:**
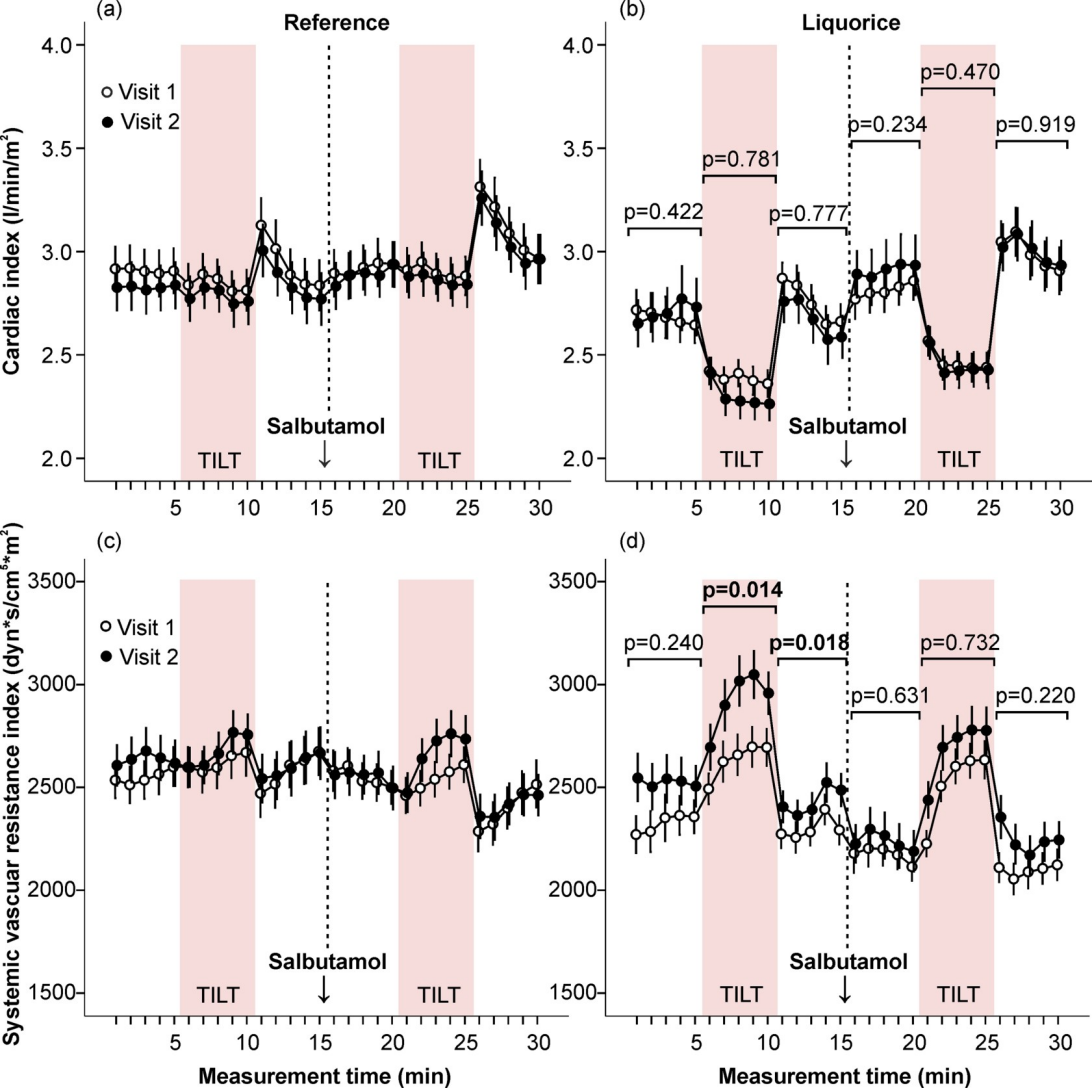
Cardiac index and systemic vascular resistance index in the absence and presence of salbutamol inhalation. Cardiac index (a, b) and systemic vascular resistance index (c, d) were measured at baseline and after the follow-up. Mean and standard error of the mean, the difference of the area under the curve between visit 1 and visit 2 was compared in the liquorice versus the reference group, adjusted for age and sex.

In the reference group, no significant changes in haemodynamics were detected during the follow-up (Figs 2 and 3 and 5–7 and S1). Two weeks of liquorice intake increased aortic systolic BP before nitroglycerin throughout the 15-min recording, and also after nitroglycerin during the orthostatic challenge and the 5-min supine period thereafter (Fig 2B). Aortic diastolic BP was increased before nitroglycerin during both 5-min supine positions, and after nitroglycerin during the final 5-min supine period (Fig 2D). The HR response to upright posture was numerically but not significantly lower after liquorice consumption (unadjusted p = 0.067 in the absence and p = 0.064 in the presence of nitroglycerin, age and sex-adjusted p = 0.291 and p = 0.225, respectively) (Fig 3C). An increase in AIx following liquorice diet was detected before nitroglycerin during the first 5-min supine period and orthostatic challenge (Fig 3D). Liquorice consumption had no significant effects on cardiac index (Fig 5B), but higher SVRI was observed before nitroglycerin during the final 5-min supine period, and after nitroglycerin during the orthostatic challenge and final 5-min in supine position (Fig 5D).

#### Liquorice and haemodynamics in the absence and presence of salbutamol inhalation

The effects of liquorice ingestion on aortic systolic and diastolic BP before and after salbutamol inhalation were corresponding to those detected before and after sublingual nitroglycerin (S1 Fig). In unadjusted analyses liquorice ingestion reduced HR response to upright posture before (p = 0.032) but not after salbutamol (p = 0.060), and when age and sex were included as covariates no significant differences were detected in cardiac chronotropic response to upright posture between the groups (Fig 6B). Liquorice intake slightly elevated AIx before and after salbutamol during two 5-min supine periods (Fig 6D). In the liquorice group, no significant changes were detected in cardiac index when compared to the reference group in the absence and presence of salbutamol (Fig 7B), while the liquorice-induced moderate increase in upright SVRI was abolished after salbutamol (Fig 7D).

## Discussion

**Fig 8:**
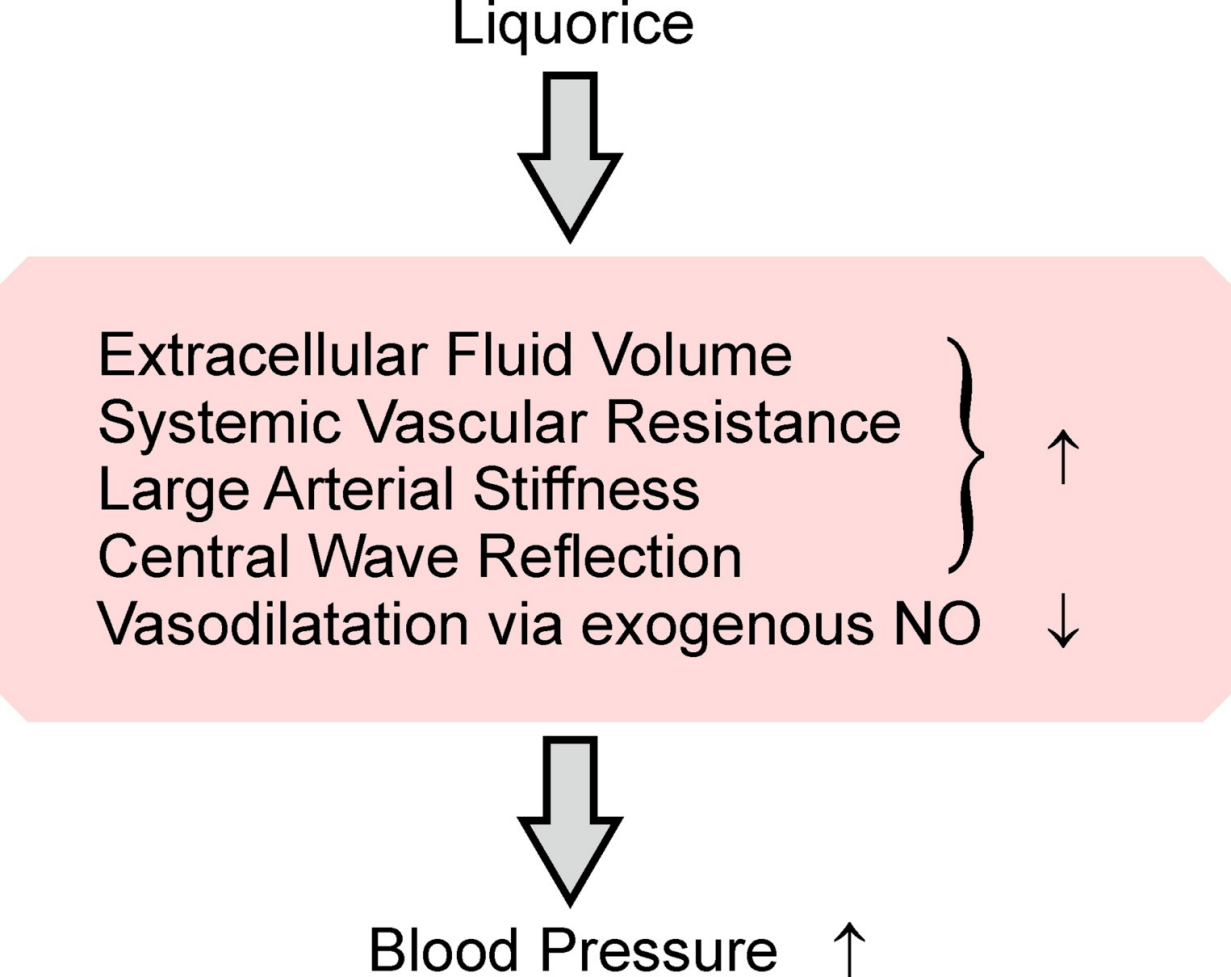
Summary of the mechanisms of liquorice-induced elevation in blood pressure.

We investigated the effects of two-week-long liquorice ingestion on the haemodynamic changes elicited by exogenous NO donor (nitroglycerin) and β_2_-adrenoceptor agonist (salbutamol) during orthostatic challenge. Liquorice exposure elevated aortic systolic and diastolic BP, and SVRI when compared with the reference group. In response to the research drug administration the liquorice-induced increase in SVRI was observed in the presence of nitroglycerin but no longer in the presence of salbutamol. Thus, liquorice intake attenuated the vasodilatory effect of exogenous NO donor, but not that of β_2_-adrenoceptor agonist. Increased extracellular water volume does not seem like the probable explanation for these findings. The present results support the view that liquorice intake elevates BP via multiple mechanisms (Fig 8).

The inhibition of enzyme 11β-HSD2 was evaluated by examining the ratio of urinary cortisone to cortisol metabolites [8]. In this study, the liquorice-induced reduction in urinary THE excretion and ratio of THE to allo-THF+THF showed that the conversion of cortisol to cortisone was impaired [8,37]. Subsequently, plasma potassium and aldosterone concentrations were decreased, and plasma renin activity was reduced, in line with previous reports [8,38]. These results suggest that MR activation was enhanced. Altered 11β-HSD activity in the vascular wall may also contribute to the elevation of BP [9]. In human VSMC, diminished 11β-HSD2 activity enhanced cortisol-induced increase in angiotensin II binding and the upregulation was inhibited by MR and GR antagonists [11]. In rats, inhibition of 11β-HSD2 induced by GA supplementation caused endothelial dysfunction, activated the ET-1 system, and elevated BP that was normalized by MR and ET_A_-receptor antagonists [39,40]. Endothelial dysfunction in 11β-HSD2 knockout mice may also result from additional indirect mechanisms related to renal sodium retention and BP elevation, and not merely from increased activation of corticosteroid receptors in the endothelium [10]. The enzyme 11β-HSD2 is co-expressed with the MR in the nucleus of the solitary tract and this might have a role in the central regulation of BP [13]. Interestingly, deficient central 11β-HSD2 activity in mice increases their appetite for salt, promotes salt-sensitivity, and enhances pressor responses to α_1_-adrenoceptor stimulation [41]. In humans, liquorice ingestion induced a greater rise in BP in hypertensive than in normotensive subjects [42]. Other factors that increase susceptibility to liquorice-induced elevation of BP are old age, female sex, hypokalaemia, prolonged gastrointestinal transit time, decreased 11β-HSD2 activity, and anorexia nervosa [1]. Sigurjonsdottir et al. found that enhanced BP response to liquorice in hypertensive subjects was not attributed to age, increased salt sensitivity or weight, and the authors suggested that increased sensitivity to the inhibition of the 11β-HSD2 was the underlying mechanism [42].

Nitroglycerin is a known exogenous NO donor and endothelium-independent vasodilator, while the β_2_-sympatomimetic salbutamol induces vasodilatation that is mediated via a direct relaxing action on VSMC and NO release from the endothelium [43,44]. Both sublingual nitroglycerin and inhaled salbutamol can reduce systemic vascular resistance in humans [22]. In the present study, two weeks of liquorice ingestion moderately elevated SVRI in the presence of nitroglycerin but not in the presence of salbutamol. This suggests that liquorice intake impaired vasodilatation via exogenous NO in vascular smooth muscle, while the β_2_-adrenoceptor-mediated vascular relaxation was not affected. In accordance with our results, Sobieszczyk et al. demonstrated that GA attenuated endothelium-independent vasodilatation in the forearm blood flow in response to verapamil, while no significant influence was observed in the endothelium-dependent vasodilatation elicited by methacholine [45]. In addition, aortas from 11β-HSD2 knockout mice demonstrated impaired vasorelaxation to both endothelium-dependent and -independent agents [46]. In contrast, endothelium-dependent but not -independent relaxation was impaired in the aortas of GA-treated Wistar-Kyoto rats [39,40]. In the present study, PWV, a marker of arterial stiffness [47], was increased after liquorice ingestion. This was probably due to the elevation of BP, as the prevailing BP that is distending the blood vessels has a significant influence on the indices of arterial stiffness [47].

The level of AIx is influenced by arterial stiffness, but also height, gender, HR, systemic vascular resistance and stroke volume contribute to the magnitude of wave reflections [28,47,48]. The changes in AIx induced by salbutamol inhalation have been utilised with the idea that they reflect alterations in the endothelium-mediated vascular tone [29,49]. However, a recent study suggested that the reduction in AIx induced by salbutamol may be largely explained by the parallel increase in heart rate [50]. In our earlier report the liquorice-induced elevation of AIx during orthostatic challenge correlated with the simultaneous reduction in HR, increase in SVRI, and prolongation of ejection duration [17]. As a novel finding, AIx remained slightly elevated following salbutamol inhalation in the supine position in spite of the parallel reduction in SVRI. However, the liquorice-induced increase in AIx was abolished in response to nitroglycerin. This can probably be explained by the powerful vasodilatory effect of nitroglycerin, as AIx level is significantly influenced by systemic vascular resistance [28]. Corresponding to previous findings, the haemodynamic effects of salbutamol were more evident in the supine position, while those of sublingual nitroglycerin were accentuated in the upright position [22,51].

Sublingual nitroglycerin and inhaled salbutamol increase HR [22], and this was also observed in the present study. Our previous report found that cardiac chronotropic response to upright posture was reduced after liquorice diet [17]. Here, liquorice intake resulted in numerically but not significantly lower upright HR throughout the recording protocol in analyses adjusted for age and sex. The present study with a small number of subjects was probably underpowered to detect significant effects of liquorice intake on the upright regulation of HR in adjusted analyses [32].

Due to the sweet taste of glycyrrhizin, liquorice extract is used as a sweetener or flavouring agent [52]. Average daily glycyrrhizin consumption in the US population was estimated to be 0.027–3.6 mg/kg (or 1.6–215 mg) [2]. During a 12-month period, ~686,000 kg of liquorice root extract was imported to Finland (customs statistics in May 2013; www.tulli.fi) [18]. This corresponds to a daily intake of ~21 mg glycyrrhizin if distributed per capita, but Finnish liquorice products are also exported [18]. A survey of 603 high school students in New Zealand indicated that 5.9% of the girls and 4.9% of the boys consumed at least 200 g of liquorice per week, while the highest consumption exceeded 1000 g per week [53]. Therefore, ingestion of glycyrrhizin products should always be considered as a putative cause for elevated BP in hypertensive patients to avoid unnecessary medical therapy.

Previously, we investigated the haemodynamic influences of liquorice in comparison with an age-matched control group [17,18]. However, repeated haemodynamic measurements with nitroglycerin and salbutamol administration were not performed in these control subjects. Therefore the results of the present study were analysed in parallel with another reference group. The reproducibility of the haemodynamic responses during head-up tilt was shown during 12 weeks of follow-up [36]. As a limitation, there were baseline differences between the present groups in demographic characteristics, laboratory values, and BP. The results were adjusted for age and sex, as these variables were considered the most relevant confounding factors influencing the haemodynamic profiles of the subjects. Aging is known to increase arterial stiffness, with subsequent increases in PWV, wave reflection, and central and peripheral systolic BP [34]. As a further sex-related difference, we previously found that the haemodynamic workload for the heart in the upright position was higher in men than in women [35]. The mean baseline brachial office BP of the reference group was high normal (134/85 mmHg), while the corresponding values in the liquorice group were optimal (119/70 mmHg) [54]. The purpose of the reference group was merely to examine whether the results of the haemodynamic recordings change during repeated measurements. Previously, liquorice ingestion has induced more pronounced clinical symptoms in women than in men, although the changes in BP were not different between the sexes [42]. However, another study suggested that men were more responsive to the liquorice-induced decrease in plasma aldosterone concentration than women [55]. Due to the small number of subjects, we did not analyse the influences of liquorice intake separately in male and female subjects. Future studies should compare the effects of gender on the influences of liquorice ingestion.

In conclusion, two weeks of liquorice ingestion resulted in the elevation of BP via multiple mechanisms. The present results suggest that liquorice exposure increased systemic vascular resistance via impaired response to NO in the vasculature, while the vasodilatory response to β_2_-adrenoceptor stimulation was not significantly altered.

## Supporting information

S1 Protocol Trial protocol. (PDF)

S2 Protocol English summary of trial protocol. (PDF)

S1 CONSORT checklist CONSORT checklist. (PDF)

S1 Fig Aortic blood pressure in the absence and presence of salbutamol inhalation. Systolic (a, b) and diastolic (c, d) blood pressure were measured at baseline and after the follow-up (reference group) or intervention (liquorice ingestion) during supine position and orthostatic challenge. Mean and standard error of the mean, statistical analyses compare the difference of the area under the curve between visit 1 and visit 2 in the liquorice versus reference group, adjusted for age and sex.
(TIF)

S1 Dataset Liquorice study demographic and clinical data. (SAV)

S2 Dataset Liquorice study haemodynamic data. (SAV)

## References

[1] Nazari S, Rameshrad M, Hosseinzadeh H. Toxicological effects of glycyrrhiza glabra (licorice): a review. Phytother Res. 2017;31: 1635–1650. 10.1002/ptr.5893 28833680

[2] Isbrucker RA, Burdock GA. Risk and safety assessment on the consumption of Licorice root (Glycyrrhiza sp.), its extract and powder as a food ingredient, with emphasis on the pharmacology and toxicology of glycyrrhizin. Regul Toxicol Pharmacol. 2006;46: 167–192. 10.1016/j.yrtph.2006.06.002 16884839

[3] Stewart PM, Wallace AM, Valentino R, Burt D, Shackleton CH, Edwards CR. Mineralocorticoid activity of liquorice: 11-beta-hydroxysteroid dehydrogenase deficiency comes of age. Lancet. 1987;2: 821–824. 10.1016/s0140-6736(87)91014-2 2889032

[4] Hunter RW, Bailey MA. Glucocorticoids and 11β-hydroxysteroid dehydrogenases: mechanisms for hypertension. Curr Opin Pharmacol. 2015;21: 105–114. 10.1016/j.coph.2015.01.005 25666420

[5] Goodwin JE, Geller DS. Glucocorticoid-induced hypertension. Pediatr Nephrol. 2012;27: 1059–1066. 10.1007/s00467-011-1928-4 21744056

[6] Funder JW, Pearce PT, Smith R, Smith AI. Mineralocorticoid action: target tissue specificity is enzyme, not receptor, mediated. Science. 1988;242: 583–585. 10.1126/science.2845584 2845584

[7] Edwards CR, Stewart PM, Burt D, Brett L, McIntyre MA, Sutanto WS, et al Localisation of 11 beta-hydroxysteroid dehydrogenase—tissue specific protector of the mineralocorticoid receptor. Lancet. 1988;2: 986–989. 10.1016/s0140-6736(88)90742-8 2902493

[8] Farese RV, Biglieri EG, Shackleton CH, Irony I, Gomez-Fontes R. Licorice-induced hypermineralocorticoidism. N Engl J Med. 1991;325: 1223–1227. 10.1056/NEJM199110243251706 1922210

[9] Hadoke PWF, Macdonald L, Logie JJ, Small GR, Dover AR, Walker BR. Intra-vascular glucocorticoid metabolism as a modulator of vascular structure and function. Cell Mol Life Sci. 2006;63: 565–578. 10.1007/s00018-005-5427-2 16416027 PMC11136224

[10] Christy C, Hadoke PWF, Paterson JM, Mullins JJ, Seckl JR, Walker BR. 11β-hydroxysteroid dehydrogenase type 2 in mouse aorta: localization and influence on response to glucocorticoids. Hypertension. 2003;42: 580–587. 10.1161/01.HYP.0000088855.06598.5B 12925564

[11] Hatakeyama H, Inaba S, Takeda R, Miyamori I. 11beta-hydroxysteroid dehydrogenase in human vascular cells. Kidney Int. 2000;57: 1352–1357. 10.1046/j.1523-1755.2000.00974.x 10760066

[12] Mahmud A, Feely J. Aldosterone-to-renin ratio, arterial stiffness, and the response to aldosterone antagonism in essential hypertension. Am J Hypertens. 2005;18: 50–55. 10.1016/j.amjhyper.2004.08.026 15691617

[13] Chapman K, Holmes M, Seckl J. 11β-hydroxysteroid dehydrogenases: intracellular gate-keepers of tissue glucocorticoid action. Physiol Rev. 2013;93: 1139–1206. 10.1152/physrev.00020.2012 23899562 PMC3962546

[14] Lombès M, Alfaidy N, Eugene E, Lessana A, Farman N, Bonvalet JP. Prerequisite for cardiac aldosterone action. Mineralocorticoid receptor and 11 beta-hydroxysteroid dehydrogenase in the human heart. Circulation. 1995;92: 175–182. 10.1161/01.cir.92.2.175 7600648

[15] Richardson RV, Batchen EJ, Denvir MA, Gray GA, Chapman KE. Cardiac GR and MR: From Development to Pathology. Trends Endocrinol Metab. 2016;27: 35–43. 10.1016/j.tem.2015.10.001 26586027

[16] Oakley RH, Cidlowski JA. Glucocorticoid signaling in the heart: A cardiomyocyte perspective. J Steroid Biochem Mol Biol. 2015;153: 27–34. 10.1016/j.jsbmb.2015.03.009 25804222 PMC4568128

[17] Hautaniemi EJ, Tahvanainen AM, Koskela JK, Tikkakoski AJ, Kähönen M, Uitto M, et al Voluntary liquorice ingestion increases blood pressure via increased volume load, elevated peripheral arterial resistance, and decreased aortic compliance. Sci Rep. 2017;7: 10947 10.1038/s41598-017-11468-7 28887501 PMC5591274

[18] Leskinen MH, Hautaniemi EJ, Tahvanainen AM, Koskela JK, Päällysaho M, Tikkakoski AJ, et al Daily liquorice consumption for two weeks increases augmentation index and central systolic and diastolic blood pressure. PloS One. 2014;9: e105607 10.1371/journal.pone.0105607 25153328 PMC4143270

[19] Rule AD, Larson TS, Bergstralh EJ, Slezak JM, Jacobsen SJ, Cosio FG. Using serum creatinine to estimate glomerular filtration rate: accuracy in good health and in chronic kidney disease. Ann Intern Med. 2004;141: 929–937. 10.7326/0003-4819-141-12-200412210-00009 15611490

[20] Turpeinen U, Markkanen H, Sane T, Hämäläinen E. Determination of free tetrahydrocortisol and tetrahydrocortisone ratio in urine by liquid chromatography-tandem mass spectrometry. Scand J Clin Lab Invest. 2006;66: 147–159. 10.1080/00365510500474504 16537248

[21] Tahvanainen A, Koskela J, Tikkakoski A, Lahtela J, Leskinen M, Kähönen M, et al Analysis of cardiovascular responses to passive head-up tilt using continuous pulse wave analysis and impedance cardiography. Scand J Clin Lab Invest. 2009;69: 128–137. 10.1080/00365510802439098 18850486

[22] Tahvanainen AM, Tikkakoski AJ, Leskinen MH, Nordhausen K, Kähönen M, Kööbi T, et al Supine and upright haemodynamic effects of sublingual nitroglycerin and inhaled salbutamol: a double-blind, placebo-controlled, randomized study. J Hypertens. 2012;30: 297–306. 10.1097/HJH.0b013e32834e4b26 22179079

[23] Chen CH, Nevo E, Fetics B, Pak PH, Yin FC, Maughan WL, et al Estimation of central aortic pressure waveform by mathematical transformation of radial tonometry pressure. Validation of generalized transfer function. Circulation. 1997;95: 1827–1836. 10.1161/01.cir.95.7.1827 9107170

[24] Kööbi T, Kaukinen S, Ahola T, Turjanmaa VM. Non-invasive measurement of cardiac output: whole-body impedance cardiography in simultaneous comparison with thermodilution and direct oxygen Fick methods. Intensive Care Med. 1997;23: 1132–1137. 10.1007/s001340050469 9434918

[25] Kööbi T, Kaukinen S, Turjanmaa VM, Uusitalo AJ. Whole-body impedance cardiography in the measurement of cardiac output. Crit Care Med. 1997;25: 779–785. 10.1097/00003246-199705000-00012 9187596

[26] Kööbi T, Kähönen M, Iivainen T, Turjanmaa V. Simultaneous non-invasive assessment of arterial stiffness and haemodynamics—a validation study. Clin Physiol Funct Imaging. 2003;23: 31–36. 12558611 10.1046/j.1475-097x.2003.00465.x

[27] Koskela JK, Tahvanainen A, Haring A, Tikkakoski AJ, Ilveskoski E, Viitala J, et al Association of resting heart rate with cardiovascular function: a cross-sectional study in 522 Finnish subjects. BMC Cardiovasc Disord. 2013;13: 102 10.1186/1471-2261-13-102 24237764 PMC3832902

[28] Wilenius M, Tikkakoski AJ, Tahvanainen AM, Haring A, Koskela J, Huhtala H, et al Central wave reflection is associated with peripheral arterial resistance in addition to arterial stiffness in subjects without antihypertensive medication. BMC Cardiovasc Disord. 2016;16: 131 10.1186/s12872-016-0303-6 27266507 PMC4897906

[29] Wilkinson IB, Hall IR, MacCallum H, Mackenzie IS, McEniery CM, van der Arend BJ, et al Pulse-wave analysis: clinical evaluation of a noninvasive, widely applicable method for assessing endothelial function. Arterioscler Thromb Vasc Biol. 2002;22: 147–152. 10.1161/hq0102.101770 11788475

[30] Tahvanainen A, Koskela J, Leskinen M, Ilveskoski E, Nordhausen K, Kähönen M, et al Reduced systemic vascular resistance in healthy volunteers with presyncopal symptoms during a nitrate-stimulated tilt-table test. Br J Clin Pharmacol. 2011;71: 41–51. 10.1111/j.1365-2125.2010.03794.x 21143500 PMC3018025

[31] Calculation of within-case area under function curve by trapezoidal integration [Internet] [cited 15 August 2019]. Available from: https://www-01.ibm.com/support/docview.wss?uid=swg21476168

[32] Streiner DL. Control or overcontrol for covariates? Evid Based Ment Health. 2016;19: 4–5. 10.1136/eb-2015-102294 26755716 PMC10699339

[33] Roberts C, Torgerson DJ. Understanding controlled trials: baseline imbalance in randomised controlled trials. BMJ. 1999;319: 185 10.1136/bmj.319.7203.185 10406763 PMC1116277

[34] McEniery CM, Yasmin, Hall IR, Qasem A, Wilkinson IB, Cockcroft JR, et al Normal vascular aging: differential effects on wave reflection and aortic pulse wave velocity: the Anglo-Cardiff Collaborative Trial (ACCT). J Am Coll Cardiol. 2005;46: 1753–1760. 10.1016/j.jacc.2005.07.037 16256881

[35] Kangas P, Tahvanainen A, Tikkakoski A, Koskela J, Uitto M, Viik J, et al Increased Cardiac Workload in the Upright Posture in Men: Noninvasive Hemodynamics in Men Versus Women. J Am Heart Assoc. 2016;5 10.1161/JAHA.115.002883 27329447 PMC4937251

[36] Hautaniemi EJ, Tikkakoski AJ, Tahvanainen A, Nordhausen K, Kähönen M, Mattsson T, et al Effect of fermented milk product containing lactotripeptides and plant sterol esters on haemodynamics in subjects with the metabolic syndrome—a randomised, double-blind, placebo-controlled study. Br J Nutr. 2015;114: 376–386. 10.1017/S0007114515002032 26168857

[37] Hammer F, Stewart PM. Cortisol metabolism in hypertension. Best Pract Res Clin Endocrinol Metab. 2006;20: 337–353. 10.1016/j.beem.2006.07.001 16980198

[38] Epstein MT, Espiner EA, Donald RA, Hughes H. Effect of eating liquorice on the renin-angiotensin aldosterone axis in normal subjects. Br Med J. 1977;1: 488–490. 10.1136/bmj.1.6059.488 837172 PMC1605097

[39] Quaschning T, Ruschitzka F, Shaw S, Lüscher TF. Aldosterone receptor antagonism normalizes vascular function in liquorice-induced hypertension. Hypertension. 2001;37: 801–805. 10.1161/01.hyp.37.2.801 11230376

[40] Ruschitzka F, Quaschning T, Noll G, deGottardi A, Rossier MF, Enseleit F, et al Endothelin 1 type a receptor antagonism prevents vascular dysfunction and hypertension induced by 11beta-hydroxysteroid dehydrogenase inhibition: role of nitric oxide. Circulation. 2001;103: 3129–3135. 10.1161/01.cir.103.25.3129 11425780

[41] Evans LC, Ivy JR, Wyrwoll C, McNairn JA, Menzies RI, Christensen TH, et al Conditional deletion of hsd11b2 in the brain causes salt appetite and hypertension. Circulation. 2016;133: 1360–1370. 10.1161/CIRCULATIONAHA.115.019341 26951843 PMC4819772

[42] Sigurjonsdottir HA, Manhem K, Axelson M, Wallerstedt S. Subjects with essential hypertension are more sensitive to the inhibition of 11 β-HSD by liquorice. J Hum Hypertens. 2003;17: 1001504 10.1038/sj.jhh.1001504 12574791

[43] Dawes M, Chowienczyk PJ, Ritter JM. Effects of inhibition of the L-arginine/nitric oxide pathway on vasodilation caused by beta-adrenergic agonists in human forearm. Circulation. 1997;95: 2293–2297. 10.1161/01.cir.95.9.2293 9142007

[44] Ferro A, Coash M, Yamamoto T, Rob J, Ji Y, Queen L. Nitric oxide-dependent β2-adrenergic dilatation of rat aorta is mediated through activation of both protein kinase A and Akt. Br J Pharmacol. 2004;143: 397–403. 10.1038/sj.bjp.0705933 15351777 PMC1575346

[45] Sobieszczyk P, Borlaug BA, Gornik HL, Knauft WD, Beckman JA. Glycyrrhetinic acid attenuates vascular smooth muscle vasodilatory function in healthy humans. Clin Sci. 2010;119: 437–442. 10.1042/CS20100087 20515440

[46] Hadoke PW, Christy C, Kotelevtsev YV, Williams BC, Kenyon CJ, Seckl JR, et al Endothelial cell dysfunction in mice after transgenic knockout of type 2, but not type 1, 11beta-hydroxysteroid dehydrogenase. Circulation. 2001;104: 2832–2837. 10.1161/hc4801.100077 11733403

[47] Laurent S, Cockcroft J, Van Bortel L, Boutouyrie P, Giannattasio C, Hayoz D, et al Expert consensus document on arterial stiffness: methodological issues and clinical applications. Eur Heart J. 2006;27: 2588–2605. 10.1093/eurheartj/ehl254 17000623

[48] Dart AM, Kingwell BA. Pulse pressure—a review of mechanisms and clinical relevance. J Am Coll Cardiol. 2001;37: 975–984. 10.1016/s0735-1097(01)01108-1 11263624

[49] McEniery CM, Wallace S, Mackenzie IS, McDonnell B, Yasmin, Newby DE, et al Endothelial function is associated with pulse pressure, pulse wave velocity, and augmentation index in healthy humans. Hypertension. 2006;48: 602–608. 10.1161/01.HYP.0000239206.64270.5f 16940223

[50] Tikkakoski AJ, Kangas P, Suojanen L, Tahvanainen AM, Eräranta A, Kähönen MAP, et al Salbutamol-induced decrease in augmentation index is related to the parallel increase in heart rate. Basic Clin Pharmacol Toxicol. 2018; 10.1111/bcpt.12988 29476697

[51] Tahvanainen A, Leskinen M, Koskela J, Ilveskoski E, Alanko J, Kähönen M, et al Non-invasive measurement of the haemodynamic effects of inhaled salbutamol, intravenous L-arginine and sublingual nitroglycerin. Br J Clin Pharmacol. 2009;68: 23–33. 10.1111/j.1365-2125.2009.03434.x 19660000 PMC2732937

[52] Omar HR, Komarova I, El-Ghonemi M, Fathy A, Rashad R, Abdelmalak HD, et al Licorice abuse: time to send a warning message. Ther Adv Endocrinol Metab. 2012;3: 125–138. 10.1177/2042018812454322 23185686 PMC3498851

[53] Simpson FO, Currie IJ. Licorice consumption among high school students. N Z Med J. 1982;95: 31–33. 6950316

[54] Williams B, Mancia G, Spiering W, Agabiti Rosei E, Azizi M, Burnier M, et al 2018 ESC/ESH Guidelines for the management of arterial hypertension. Eur Heart J. 2018;39: 3021–3104. 10.1093/eurheartj/ehy339 30165516

[55] Sigurjonsdottir HA, Axelson M, Johannsson G, Manhem K, Nyström E, Wallerstedt S. The liquorice effect on the RAAS differs between the genders. Blood Press. 2006;15: 169–172. 10.1080/08037050600593060 16864159

