# Supplementary figures and images for: Liquorice ingestion attenuates vasodilatation via exogenous nitric oxide donor but not via β2-adrenoceptor stimulation
Source: PLoS One. 2019 Oct 18;14(10):e0223654. doi: 10.1371/journal.pone.0223654 (PMC6799927; doi:10.1371/journal.pone.0223654)

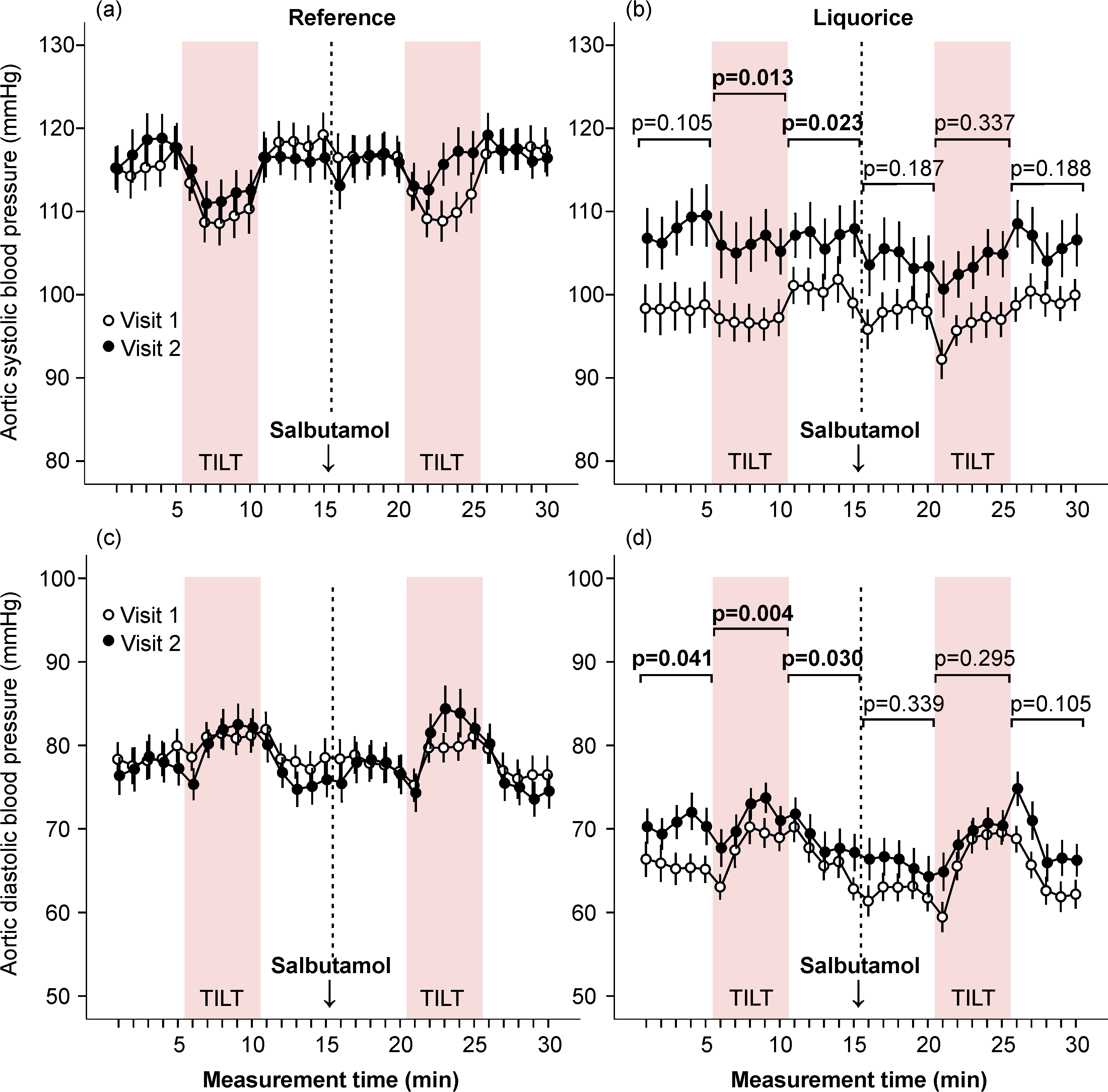

Supplement: S1 Fig — Systolic (a, b) and diastolic (c, d) blood pressure were measured at baseline and after the follow-up (reference group) or intervention (liquorice ingestion) during supine position and orthostatic challenge. Mean and standard error of the mean, statistical analyses compare the difference of the area under the curve between visit 1 and visit 2 in the liquorice versus reference group, adjusted for age and sex. (TIF) [file pone.0223654.s004.tif]
